# Medical College in Detroit

**Published:** 1868-05

**Authors:** 


*Medical College in Detroit.*—The physicians connected with the Harper Hospital and the public-spirited and wealthy citizens of the city, are engaged in the establishment of the Detroit Medical College. The enterprize will doubtless prove a success, as those engaged in the effort are earnest and successful in their undertakings. Its professorship will, we learn, be filled for the most part by Detroit men. Dr. S. G. Armor, Dr. E. W. Jenks, Dr. T. A. McGraw, Dr. G. P. Andrews, Dr D. O. Farrand and Dr. S. P. Duffield, are all spoken of in connection with certain departments of instruction. Other resident physicians will probably be invited to fill chairs in the college.

